# Current landscape in antiviral drug development against herpes simplex virus infections

**DOI:** 10.1002/SMMD.20220004

**Published:** 2022-12-16

**Authors:** Fanny Frejborg, Kiira Kalke, Veijo Hukkanen

**Affiliations:** ^1^ Pharmaceutical Sciences Laboratory Faculty of Science and Engineering Åbo Akademi University Turku Finland; ^2^ Institute of Biomedicine Faculty of Medicine University of Turku Turku Finland

**Keywords:** antiviral therapies, clinical trials, drug development, herpes simplex virus, HSV, infections

## Abstract

Herpes simplex viruses (HSV) are common human pathogens with a combined global seroprevalence of 90% in the adult population. HSV‐1 causes orofacial herpes but can cause severe diseases, such as the potentially fatal herpes encephalitis and herpes keratitis, a prevalent cause of infectious blindness. The hallmark of HSV is lifelong latent infections and viral reactivations, leading to recurrent lesions or asymptomatic shedding. HSV‐1 and HSV‐2 can cause recurrent, painful, and socially limiting genital lesions, which predispose to human immunodeficiency virus infections, and can lead to neonatal herpes infections, a life‐threatening condition for the newborn. Despite massive efforts, there is no vaccine against HSV, as both viruses share the capability to evade the antiviral defenses of human and to establish lifelong latency. Recurrent and primary HSV infections are treated with nucleoside analogs, but the treatments do not completely eliminate viral shedding and transmission. Drug‐resistant HSV strains can emerge in relation to long‐term prophylactic treatment. Such strains are likely to be resistant to other chemotherapies, justifying the development of novel antiviral treatments. The importance of developing new therapies against HSV has been recognized by the World Health Organization. In this review, we discuss the current approaches for developing novel antiviral therapies against HSV, such as small molecule inhibitors, biopharmaceuticals, natural products, gene editing, and oligonucleotide‐based therapies. These approaches may have potential in the future to answer the unmet medical need. Furthermore, novel approaches are presented for potential eradication of latent HSV.

## Graphical abstract

Investigational therapies currently in the development for treatment of herpes simplex virus infections are reviewed, and the clinical pipeline is analyzed. These therapies include small molecule inhibitors, biopharmaceuticals, natural products, gene editing, and oligonucleotide‐based therapies. These therapies have the potential to answer the unmet medical need either by treating infections caused by acyclovir resistant herpes simplex virus strains or potentially eradicating the latent virus from the host.

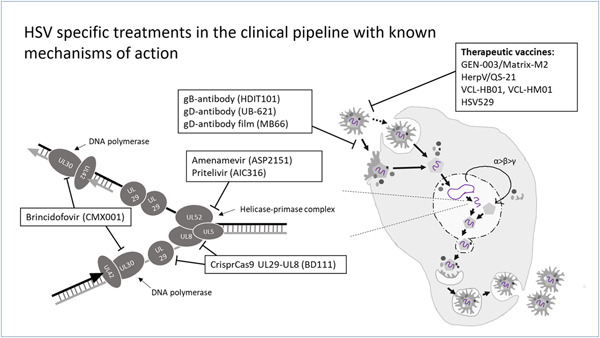

1

**Key points**

- Herpes simplex viruses (HSV) cause life‐long recurring infections that pose an unmet medical need.
- No vaccine is approved for prevention of HSV infections.
- No current treatment can eliminate the latent virus from the host. Acyclovir and drugs alike are used for acute episodes. Drug resistant strains arise during long prophylactic treatments.
- The current clinical landscape of antiviral development against HSV consist of small molecule inhibitors, biopharmaceuticals (antibodies and therapeutic vaccines), natural products, gene editing, immunomodulatory therapies and antisense oligonucleotides.

**Abbreviations**
AAP: arginine attenuator peptide
AAV: adeno‐associated virus
ACV: Acyclovir
AMP: antimicrobial peptide
ANP: atrial natriuretic peptide
ASO: antisense DNA oligonucleotide
CRISPR/Cas9: clustered regularly interspaced short palindromic repeats/CRISPR‐associated protein 9
EMA: European Medicines Agency
FDA: The United States Food and Drug Administration
gB, gD, gH/gL: viral glycoproteins (g) B, D, H/L
HSV: herpes simplex virus
ICP: infected cell protein
ICTRP: International Clinical Trials Registry Platform
miRNA: micro RNA
SADBE: squaric acid dibutyl ester
siRNA: short interfering RNA

## 2 INTRODUCTION

Herpes simplex viruses (HSV) are important pathogens that cause latent, lifelong infections in humans. There are two herpes simplex viruses, HSV‐1 and HSV‐2, which are both common viruses with global seroprevalences of 67%^1^ and 11.3%,^2^ respectively. Though HSV‐1 is known for causing orofacial infections and HSV‐2 for causing genital infections, HSV‐1 can also cause genital HSV infections. In fact, in developed countries, such as Finland, HSV‐1 has already become more prevalent than HSV‐2 as the major cause of genital herpes among young women.^3^

HSV infections are characterized by the ability of the virus to cause latent infections and subsequently reactivate, causing recurrent lesions or blisters, which are the most common symptoms of HSV infections. However, the infection may also reactivate or establish elsewhere, such as in the cornea of the eye, causing herpes simplex keratitis, the most prevalent cause of infectious blindness in developed countries,^4^ or in the brain, causing herpes simplex encephalitis, a disease with a high rate of mortality of up to 30% even with treatment.^5^ Another severe condition caused by HSV is the life‐threatening neonatal herpes, where the mother transmits, usually during childbirth, the virus to the newborn. In addition, genital HSV infections also predispose to human immunodeficiency virus infections.^6^

Vaccine development against HSV is very challenging, as the virus is capable of evading the immune system, and has been unsuccessful to date despite massive efforts.^7^ There is no cure available which would eliminate HSV from the human body, and although the current first‐in‐line treatment with acyclovir (ACV) and its derivatives can control the infection, drug‐resistant HSV strains may emerge. The resistant strains can appear in both immunocompromised and immunocompetent individuals, although the risk is higher in immunocompromised individuals. The emergence of resistant strains is especially likely in long‐term prophylactic treatment with ACV, such as is required in herpes keratitis.^8^ The ACV‐resistant strains can consequently cause multiple recurrences, which can lead to dangerous exacerbations, as these strains are often resistant to other nucleoside‐based chemotherapies as well.

The lack of treatment for ACV‐resistant strains justifies the development of new HSV therapies. The World Health Organization as well as Herpes Cure Advocacy both recognize this unmet medical need.

The development of new therapies has shifted constantly in the United States and Europe, and currently many different approaches exist. Drug development has its roots in products derived from nature. Chemically synthesized small molecules are favored currently. High‐throughput screening of molecules that can bind to targets has made finding potential candidates more streamlined. Recently, approaches, such as biopharmaceuticals and gene editing, have become popular with a lot of potential. In this review, we will discuss how these different approaches are utilized in the current landscape of developing treatments against HSV infections.

## 3 BIOLOGY AND REPLICATION OF HERPES SIMPLEX VIRUSES

**FIGURE 1:**
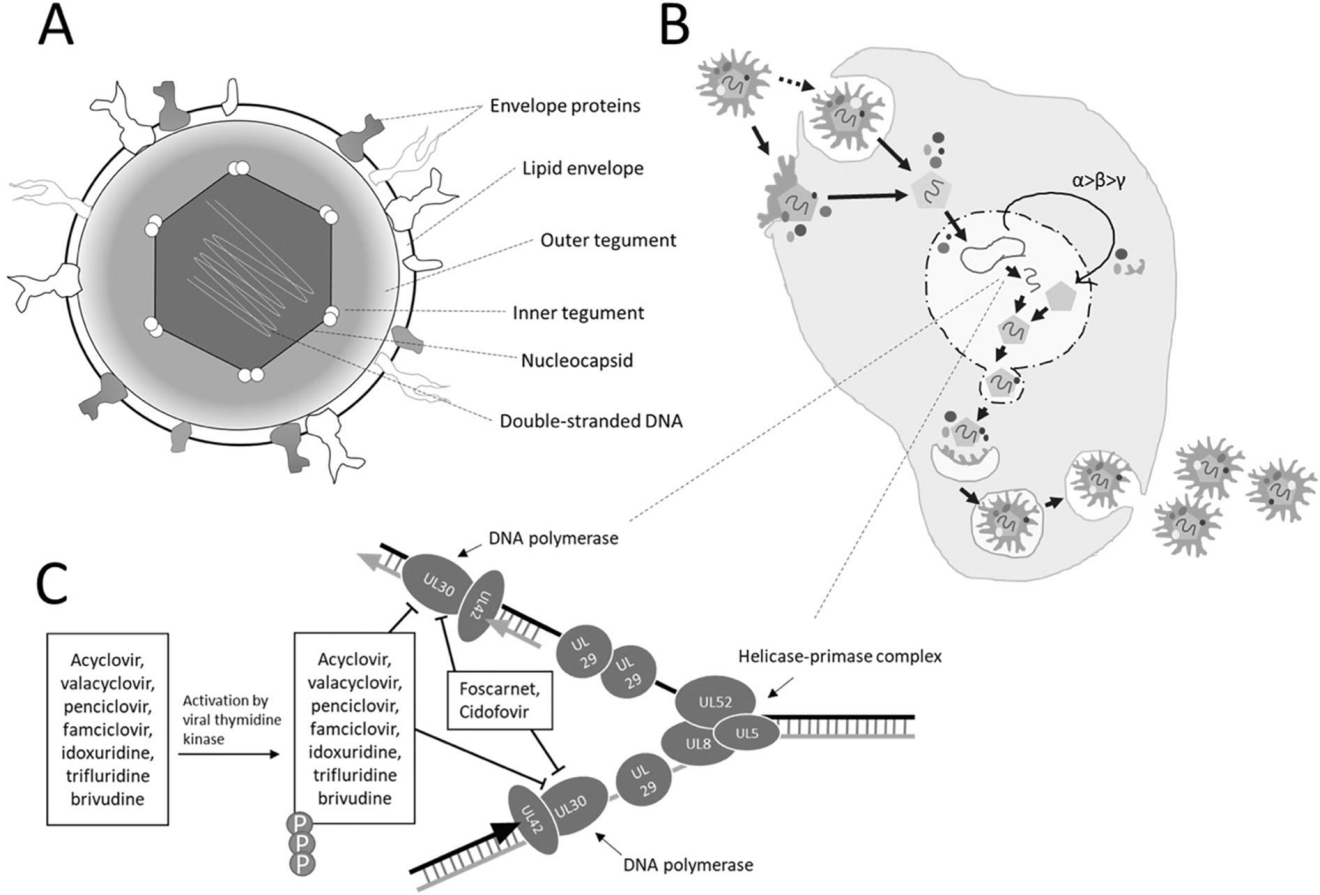
(A) Virion structure. The HSV virion consists of double‐stranded DNA encapsulated in a nucleocapsid. The nucleocapsid is surrounded by the inner and outer tegument. Surrounding the tegumented nucleocapsid is a lipid envelope with proteins. (B) HSV replication. Upon entry into the host cell, the tegument and nucleocapsid are released into the cytoplasm. The nucleocapsid releases the viral DNA into the host cell nucleus, where viral gene expression is initiated in a cascade‐like manner, enabling the viral DNA replication. New viral particles are then assembled and released from the host. (C) Mechanism of action of the current approved therapies.

HSV virions consist of double stranded DNA, a nucleocapsid, a tegument with inner and outer layers, as well as a lipid bilayer, referred to as the envelope (Figure 1A). The HSV genome has a size of approximately 152,000 base pairs and contains two unique segments with surrounding inverted repeats. The genome can exist in four different isomeric forms, depending on the orientation of the unique sequences.^9^ In the virion, the genome is contained in the icosahedral nucleocapsid in a high‐pressure state.^10^ The HSV nucleocapsid is circa 125 nm in size.^11^ Surrounding the nucleocapsid is the tegument, housing at least 20 proteins.^12^ This tegumented nucleocapsid is contained in the lipid bilayer envelope, which harbors the known 12 envelope surface glycoproteins. The envelope and its glycoproteins are essential for viral entry into the host cell.

HSV binds to the host cell via the interaction of host surface proteins and viral envelope glycoproteins. The viral entry takes place when the lipid bilayer fuses with the host membrane, the interactions requiring the viral glycoproteins gB, gD, and gH/gL, or via endocytosis (Figure 1B).^13^ After entry, HSV releases the tegumented nucleocapsid into the cytoplasm. The nucleocapsid is transported to the host nuclear pore via the host cytoskeleton, where it releases the viral DNA into the nucleus.^14^ The viral DNA does not integrate into the host genome, but persists in an episomal form.^15^ The viral transcription is executed in a cascade‐like manner.^16^ The gene products accumulate in specific sites of the cells,^17^ such as nucleocapsid proteins that assemble in the nucleus, and form new capsids that incorporate the viral DNA. The nucleocapsid is initially enveloped by the nuclear membrane and buds to the cytoplasm, and association with tegument proteins occurs either prior to nuclear egress or shortly thereafter. The cellular microtubuli are reorganized to transport the nucleocapsids to the organelles where they acquire their mature envelope.^18^ The identity of the organelle is unclear, but evidence suggests the trans‐golgi network is an important site. Once mature, the HSV particle egresses from the host. After the egress of the mature virions, the host cell is destroyed, and the virions proceed to infect the next cells either apically or laterally. The virions may also proceed via retrograde transport to sensory ganglia, where they can establish lifelong latent infections, from which the virus may then reactivate and a new lytic infection can begin.^19^

## 4 CURRENT APPROVED THERAPIES

The current treatments available for HSV infection are either nucleoside analogs, which activate via phosphorylation by the viral thymidine kinase to inhibit the viral DNA polymerase, or direct inhibitors of the viral DNA polymerase (Table 1; Figure 1C). The first‐in‐line treatment of HSV infections is acyclovir and its prodrug valaciclovir. Alternatively, penciclovir or its prodrug famciclovir can be used. The antiviral action of ACV, and of the other nucleoside analogs, depends on its phosphorylation, which occurs first by the viral thymidine kinase (Figure 1C). ACV is phosphorylated further by cellular kinases into acyclovir triphosphate, which can effectively inhibit the viral DNA polymerase, with high affinity toward the enzyme. This terminates viral DNA synthesis and thus inhibits viral replication. Consequently, this first‐in‐line therapy can only treat actively replicating HSV and requires a functional thymidine kinase for activity. The emergence of ACV‐resistant strains by mutation in the viral thymidine kinase causes cross‐resistance to all of the nucleoside analogs.

**TABLE 1:** Current approved therapies of herpes simplex virus infections

Active ingredient	Mechanism of action
Acyclovir	Converted by thymidine kinase—DNA polymerase inhibitor
Valaciclovir (prodrug of acyclovir)	Converted by thymidine kinase—DNA polymerase inhibitor
Penciclovir	Converted by thymidine kinase—DNA polymerase inhibitor
Famciclovir (prodrug of penciclovir)	Converted by thymidine kinase—DNA polymerase inhibitor
Foscarnet	DNA polymerase inhibitor
Cidofovir	DNA polymerase inhibitor

Recently, foscarnet, a second‐in‐line treatment, has become valuable in treating patients with acyclovir‐resistant viral strains. Foscarnet directly inhibits the viral DNA polymerase without prior activation by other viral proteins, allowing its efficacy against viral strains with thymidine kinase mutations. However, foscarnet can exhibit numerous adverse effects, some of which are so severe that patients are recommended to be clinically monitored. Furthermore, mutations in either or both the thymidine kinase and DNA polymerase genes are highly possible, as the genes have high variability among strains of HSV.^20^ DNA polymerase mutations can lead to simultaneous foscarnet‐ and ACV‐resistant strains, which have already been described in both immunocompromised^21^ and immunocompetent patients.^22^ Moreover, in a study, over half of bone marrow transplant patients with ACV resistance were also resistant to foscarnet.^23^ Such ACV‐ and foscarnet‐resistant viral strains can be treated with cidofovir, which is otherwise used for treatment of cytomegalovirus in immunocompromised patients. Cidofovir, however, has dangerous adverse effects, such as high renal toxicity, and is thus only used when other treatments fail, and when treatment is necessary.

## 5 CURRENT LANDSCAPE

**FIGURE 2:**
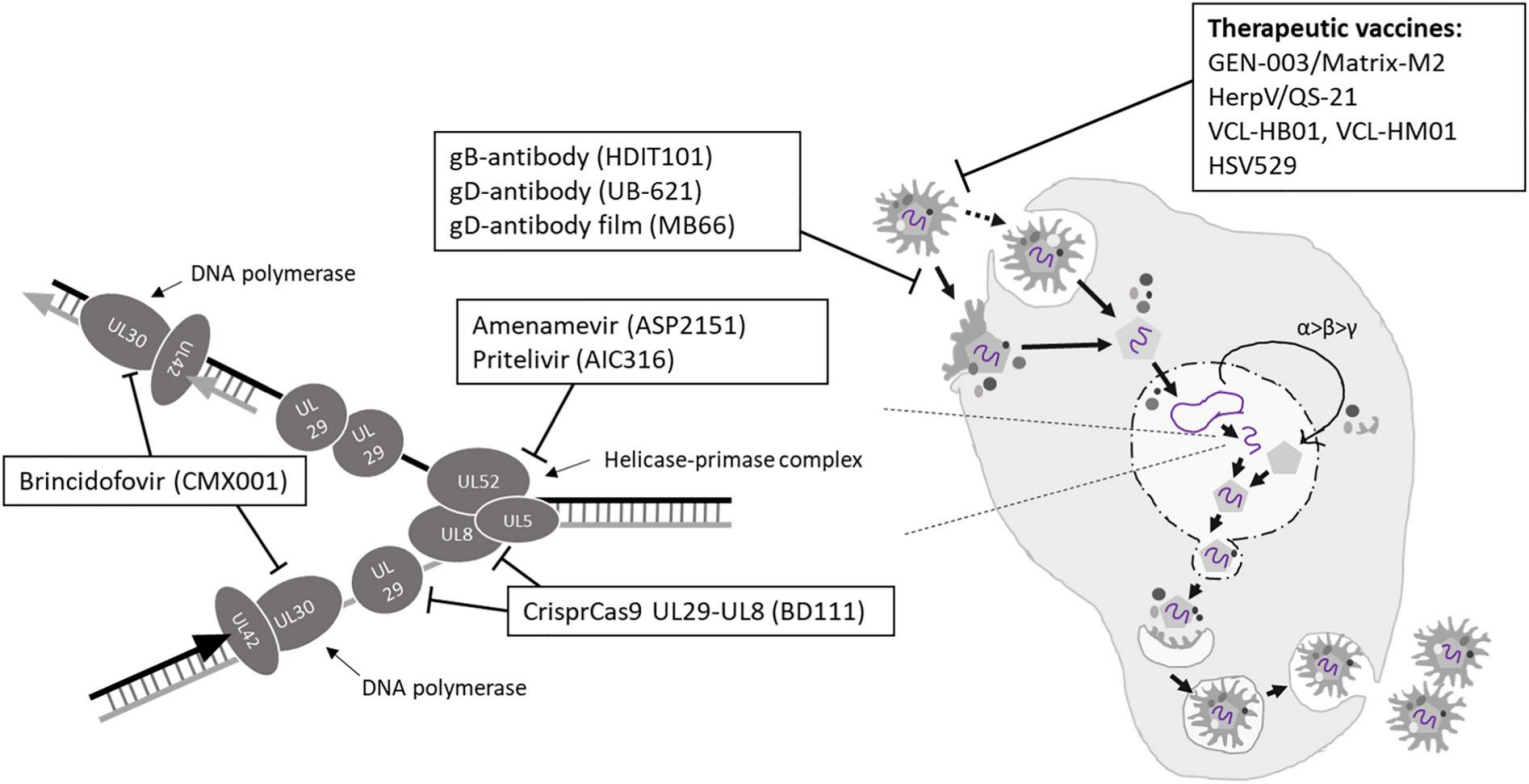
Herpes simplex virus‐specific treatments in the clinical pipeline with known mechanisms of action.

To answer the unmet medical need, many compounds targeting HSV infection are being developed. In addition to the numerous in vitro and in vivo studies on possible future treatment modalities, many compounds have already reached the clinical development phase (Table 2). The modes of action of compounds with specified targets are presented in Figure 2.

**TABLE 2:** The pipeline for HSV treatment (as of February 1, 2022)

Approach	Drug	Described indication	Treatment approach	Phase	Identifier
Small molecule inhibitors	Amenamevir (ASP2151)	Labial, facial or genital herpes simplex episodes	Inhibition of viral helicase‐primase (oral)	III	NCT01959295
					NCT02209324
	Pritelivir (AIC316)	Labial HSV	Inhibition of viral helicase‐primase (topical)	II	NCT02871492
		Acyclovir‐resistant mucocutaneous HSV	Inhibition of viral helicase‐primase (oral)	III	NCT03073967
		Recurrent genital HSV‐2		II	NCT01047540, NCT01658826
	Brincidofovir (CMX001)	Neonatal HSV infection with CNS disease	Prodrug of cidofovir, inhibition of viral polymerase and inhibition of viral DNA synthesis (oral)	I/II	NCT01610765
Gene editing	Gene editing therapy (BD111)	Refractory herpetic viral keratitis	CRISPR/Cas9 mRNA (corneal injection)	I/II	NCT04560790
Biopharmaceuticals/therapeutic vaccines	Monoclonal gB‐antibody (HDIT101)	Recurrent orolabial HSV‐1	Monoclonal type‐common gB–antibody (topical)	II	DRKS00014678, NCT04539483
					EUCTR2020‐000926‐24‐DE
		Recurrent genital HSV‐2	Monoclonal type‐common gB‐antibody (intravenous)	II	NCT04165122
	Monoclonal gD‐antibody (UB‐621)	HSV infection	Monoclonal type‐common gD‐antibody (subcutaneous)	I	NCT02346760
		Recurrent genital herpes (HSV‐2)		II	NCT04714060, NCT04979975, NCT03595995
	Monoclonal antibody film (MB66)	Reduction of vaginal transmission of HSV‐2 and HIV	Monoclonal antibody film, type‐common gD‐antibody and HIV‐antibody (topical)	I	NCT02579083
	GEN‐003, matrix‐M2	Genital herpes simplex type 2	Recombinant T cell antigens ICP4 and gD2, adjuvant (intramuscular injection)	II	NCT02515175, NCT02114060, NCT01667341, NCT02300142, NCT03146403
	HerpV, QS‐21	Genital herpes simplex type 2	Recombinant human heat shock protein 70 polyvalent peptide complexed with 32 synthetic HSV‐2 peptides, adjuvant (subcutaneous injection)	II	NCT01687595
	VCL‐HB01, VCL‐HM01	Genital herpes simplex type 2	Plasmid DNA (intramuscular injection)	I/II	NCT02837575, NCT02030301
	HSV529	Genital herpes simplex type 2	Non‐replicative HSV‐2 (injection)	I	NCT02571166
	Cryopreserved amniotic membrane (prokera slim)	Herpes simplex dendritic keratitis	Anti‐inflammatory, anti‐scarring and antiangiogenic (topical)	NA	NCT04598282
Natural products	Kanuka medical grade honey	Herpes simplex labialis	Lesion healing (topical)	NA	ACTRN12615000648527
	*Hypericum perforatum*, *Calendula officinialis* and 5% copper sulfate (dynamiclear)	Herpes simplex labialis	Lesion healing (topical)	NA	ACTRN12618000890235
	Neem based lip balm	Herpes simplex labialis	Lesion healing (topical)	I	NCT00985335
	BOR15001L7	Herpes simplex labialis	Lesion healing (topical)	II	NCT03977792, NCT02582086
	Botulinum toxin type A	Herpes simplex labialis	Prophylaxis (intramuscular injection)	NA	NCT01225341
	ZEP‐3	Herpes simplex labialis	Lesion healing (topical)	II	NCT02483182
Immunomodulatory	Squaric acid dibutyl ester (SADBE)	Prevention of recurrent herpes labialis	Enhancement of immune system (topical)	II	NCT02965781
	Dexamethasone (corticosteroid)	Herpes simplex encephalitis related cognitive defects	Anti‐inflammatory, adjuvant for standard care (intravenous)	III	NCT03084783
	Diclofenac, lidocaine (RMN3001)	Herpes labialis related pain	Anti‐inflammatory (topical)	II	NCT02207881
Other	Photodynamic therapy	Herpes labialis	Photodynamic therapy	NA	NCT04037475
	NB‐001	Recurrent HSV labialis	Positively charged particles, multiple ingredients, disruption of the viral envelope	III	NCT01695187

*Note*: Clinical trials registered in clinicaltrials.gov after 2010 are included.

Abbreviation: HSV, herpes simplex virus.

We have reviewed the current pipeline by focusing on different approaches utilized in drug development. This review focuses mostly on therapies in clinical development and clinical trials post‐2010. However, older clinical trials that have been scientifically published have also been included. This review also highlights novel areas in the development of antiviral therapies against HSV infections, such as approaches to eliminate the latent virus. We have divided our compounds of interest into subcategories, which are small molecule inhibitors, biopharmaceuticals that include therapeutic vaccines, natural products, gene editing, immunomodulatory therapies, and beyond the clinical pipeline, antisense oligonucleotide therapy.

### 5.1 Small molecule inhibitors

Small molecule inhibitors, which can target any step of the viral replication cycle, make up a majority of all antiviral drugs. Similarly, the majority of the current HSV drugs are small molecule inhibitors targeting viral DNA replication via inhibition of the viral DNA polymerase (Figure 1C). Target‐based screening is a popular approach in which inhibitors can be screened for distinct targets, and by targeting specific complexes or proteins essential for viral replication, the active replication of the virus can be terminated. This has led to small molecule inhibitors being investigated in the clinical pipeline.

In the current clinical pipeline (Table 2), there are three different small molecule inhibitors, including Brincidofovir (CMX001), a prodrug of cidofovir, and two helicase‐primase inhibitors. These two helicase‐primase inhibitors have a novel mechanism of action targeting the viral helicase‐primase complex. The complex, consisting of UL5 helicase, UL8 cofactor, and UL52 primase, is essential for viral DNA replication. Such a complex is not normally present in eukaryotic cells, meaning that in contrast to the nucleoside analogs, helicase‐primase inhibitors may selectively target the virus.

The two helicase primase‐inhibitor drug candidates are named pritelivir (AIC316) and amenamevir (ASP2151). Pritelivir has shown success in its phase II trial with better efficacy compared to valacyclovir over 4 weeks in reducing genital viral shedding^24^ and no reported emergence of resistance after 4 weeks of daily therapy.^25^ Furthermore, it has been reported efficient against an acyclovir‐resistant infection of an immunosuppressed patient.^26^ The recruitment for a phase III trial for the treatment of ACV‐resistant mucocutanous HSV infections of immunocompromised individuals is now ongoing. Amenamevir has had success in its two Japan‐based phase III studies with reported significance in lesion healing time.^27^ Hence, the company has filed for indication for amenamevir to be used for the treatment of recurrent herpes simplex infections in Japan.

Interestingly, HSV variants resistant to helicase‐primase inhibitors, including both pritelivir and amenamevir, have been reported.^28^ The mutations leading to resistance have been identified especially in UL5.^28^ ^b,c,^ ^29^ These helicase‐primase inhibitor‐resistant variants have had differences in viral growth, but a resistance allowing mutation that did not affect the viral growth nor virulence at all has been identified.^30^

### 5.2 Biopharmaceuticals and therapeutic vaccines

One of the fastest developing fields in medicine are biopharmaceuticals—compounds that are derived from living organisms. The concept of biopharmaceuticals commonly refers to monoclonal antibodies; however, biopharmaceuticals also include products, such as stem cells and tissues, vaccines, as well as gene therapy, though we will discuss gene therapy separately in this review. While there are no biopharmaceuticals approved for the treatment of HSV infections by the European Medicines Agency (EMA) or The United States Food and Drug Administration (FDA), this field is very active, particularly in investigating monoclonal antibodies and therapeutic vaccines. Monoclonal antibodies can neutralize viruses by directly binding to them, preventing the attachment to cell surfaces. However, HSV poses a difficult target to therapeutic antibodies, as it can evade antibodies through cell‐to‐cell spreading and by interfering with antibody‐dependent cellular cytotoxicity.

The adaptive host defenses against HSV infections are humoral and cell‐mediated immunity, which can limit the extent of the infection and eliminate local lytic infections. There is also evidence that responses to pre‐existing HSV infections can protect against the acquisition of a different HSV type.^31^ Neonates infected with HSV also have better protection against the disease if they have acquired maternal antibodies.^32^ However, this protection is not complete. There is evidence that HSV‐2 is more difficult to neutralize than HSV‐1.^33^ Furthermore, there is evidence that the cell‐to‐cell spreading of the virus enables it to escape serum neutralization.^34^ Yet, multiple in vivo studies show that antibodies administered post infection can decrease mortality significantly in studies on mice or guinea pigs.^35^

Multiple phase 2 trials have recently been registered, that are not yet recruiting, which will investigate subcutaneous administration of human IgG1 monoclonal antibody UB‐621 for the treatment of genital HSV‐2 infections. This antibody targets the type‐common epitopes of the surface glycoprotein D of HSV.^36^ The phase 1 trial results have not been posted, but the investigators claimed that it was well tolerated. Similarly, two phase II studies are registered and one completed with no results published, investigating a humanized IgG1 monoclonal antibody HDIT101 against HSV infections. These studies will test topical and intravenous administration against both HSV‐1 and HSV‐2 and target type‐common epitopes of the glycoprotein B.^37^ The phase I study has been completed with no results posted. Furthermore, a phase I study of a monoclonal antibody film MB66 for the reduction of vaginal transmission of HSV‐2 and HIV has recently been completed.^38^ The film contains the antibodies HSV8‐N, which binds both HSV types, and VRC01‐N, which neutralizes HIV. The film was found to be safe for humans.

Administered antibodies are not the only humoral therapy available. Therapeutic vaccines utilize immunization and development of antibodies. These vaccines are based on antigens or attenuated viruses. Several therapeutic vaccines against HSV infections have been investigated clinically. These therapeutic vaccines are indicated for use in patients already suffering from HSV infection, as opposed to prophylactic vaccines, which are administered to HSV seronegative individuals. Prophylactic vaccines will not be discussed in this review; instead, vaccines indicated as therapeutic or investigating efficacy in seropositive patients will be discussed. One such vaccine against genital HSV‐2, GEN‐003, has been studied extensively. It comprises two recombinant T cell antigens: internal fragment of the immediate early protein infected cell protein 4 (ICP)4 and glycoprotein D2, with a matrix‐M2 adjuvant, consisting of *Quillaja saponins*, phosphatidylcholine, and cholesterol. The results have been posted for a phase 2 study of GEN‐003 (NCT03146403), but the study was terminated due to a business decision. However, GEN‐003 has now been acquired by another company. Another vaccine for the treatment of genital HSV‐2 is HerpV, a recombinant human heat shock protein 70 polyvalent peptide complexed with 32 synthetic HSV‐2 peptides, and the adjuvant QS‐21. The results for this phase 2 trial have been posted with adverse effects included. VCL‐HB01 and VCL‐HM01 are two plasmid DNA vaccines against genital HSV‐2. Both of these vaccines encode two undisclosed HSV proteins. The studies are completed with no results posted. HSV529 is a non‐replicative HSV‐2 vaccine virus, from which UL5 and UL29 have been deleted. It was investigated in a phase 1 study, but no results have been posted. An active but not yet recruiting trial investigating the safety of an HSV‐2 vaccine, with undisclosed formulation, against genital HSV‐2 is registered. There are also many promising vaccines in preclinical investigation, such as a trivalent nucleoside‐modified mRNA vaccine against genital herpes.^39^

Apart from monoclonal antibodies, a trial for a physical treatment for herpes simplex dendritic keratitis is currently recruiting (NCT04598282). The Prokera Slim membrane is a cryopreserved amniotic membrane that is FDA approved as a medical device for use on ocular surfaces.

### 5.3 Natural products

Many impactful medicines approved by the FDA and EMA have their origins in nature. These have been derived from plants, animals, and fungi. Products that are derived from nature differ from chemically synthesized compounds. These differences include molecules that are complex, synergistic mixtures of biologically active compounds, and a history of traditional use. However, these products offer challenges—such as difficulty in separating compounds, incompatibility and interference with biological assays, exploitation of limited natural resources, and off‐target effects and toxicity. Many of these challenges can be overcome by modifying and chemically synthesizing the natural compound. Currently, no natural products are FDA or EMA approved for the treatment of HSV infections.

The landscape of natural derived products being investigated for antiviral potential is vast. Some of the first antiviral compounds derived from nature were the nucleosides spongothymidin and spongouridin, derived from the marine sponge *Tectitethya crypta*.^40^ These were developed into vidarabine, the first approved HSV treatment (now withdrawn).^41^ Plant^42^ and algae^43^ extracts are popular in the current landscape (Table 2). Furthermore, single compounds extracted from organisms are also extensively studied, including polysaccharides,^44^ flavonoids,^45^ alkaloids,^46^ polyphenols,^47^ anthraquinones,^48^ proteins, and peptides^49^ (Table 3). The origins for these compounds are not only plants, but can also be fungi^50^ or animals.^51^ However, apart from vidarabine, none of these compounds have been investigated clinically. Furthermore, the mechanism of action can be difficult to determine for such compounds.

**TABLE 3:** Examples of natural products investigated in in vivo HSV infection models

Active ingredients	Origin	Compound class	Model	Treatment	Results
Hydroethanolic root extract	*Tanacetum parthenium*	Crude extract	Dermal inoculation of HSV‐1 in mice	Oral and topical multiple dose treatment	The extract was safe in mice, and lesion healing was statistically significant compared to the untreated group^42^
Resveratrol	*Vitis vinifera*	Polyphenol	Vaginal inoculation of HSV‐2 in mice	Intravaginal multiple dose treatment	Statistically significant reduction in HSV‐2 replication day 1, 3 and 5 post infection^47^
GFAHP	*Grifola fondosa*	Protein	Corneal inoculation of HSV‐1 in mice	Topical multiple dose treatment	GFAHP was safe in mice, disease development was similar to untreated mice, but viral replication was statistically significantly reduced compared to acyclovir treatment^50^
ANP and AAP	*Acanthopanax sciadophylloides*	Polysaccharides	Vaginal inoculation of HSV‐2 in mice	Intravaginal multiple dose treatment	Statistically significant reduction of HSV‐2 replication day 3 post infection in mice treated with ANP or AAP compared with untreated mice, significant prolonged survival period in AAP treated mice^44^
Houttuynoid A	*Houttuynia cordata* Thunb.	Flavonoid	Dermal inoculation of HSV‐1 in mice	Topical single dose treatment	Lesion healing faster in treated mice, statistical significant reduction in HSV‐1 DNA in skin samples in treated mice at all time points^45^
CHCl_3_‐soluble alkaloid fraction from MeOH extract	*Stephania cepharantha*	Major alkaloid	Dermal inoculation of HSV‐1 in mice	Oral multiple dose treatment	The extract delayed onset of lesions, and mortality reduced, however the extract exhibited high toxicity in mice^46^
Methanol extract	*Symphyocladia latiuscula*	Crude extract	Dermal inoculation of HSV‐1 in mice	Oral multiple dose treatment	Statistically significant reduction of viral replication in treated mice compared with untreated mice^43^
Meliacine	*Melia azedarach* L.	Glycopeptide	Vaginal inoculation of HSV‐2 in mice	Intravaginal multiple dose treatment	Delay in onset of lesions in treated mice, prolonged survival period, statistically significant reduction in HSV‐2 replication on day 1, 2 and 3 post infection^49^
AMPs 1002, 1006, 1018 and HH‐2	Analogs to peptides from *Bovinae*	Peptide	Vaginal inoculation of HSV‐2 in mice	HSV‐2 inoculation mixed with AMPs; prophylactic topical single dose treatment	Significantly reduced mortality rate, viral replication and delayed disease progression in mice inoculated with HSV‐2 admixed with AMPs than mice inoculated with HSV‐2 alone^51^
Emodin	*Rheum tanguticum*	Anthraquinone	Intracerebral inoculation of HSV‐1 and HSV‐2 in mice	Oral multiple dose treatment	Increased survival rate, significant reduction in viral titers in organ homogenates compared to untreated mice^48^

Abbreviations: AAP, arginine attenuator peptide; AMP, antimicrobial peptide; ANP, atrial natriuretic peptide.

A trial evaluating the efficacy of topical treatment of kanuka medical grade honey versus 5% acyclovir cream for herpes simplex labialis has recently been completed, registered at the WHO International Clinical Trials Registry Platform (ICTRP).^52^ The trial was a randomized open‐label superiority trial where the subjects reported the progression of blisters during treatment. The honey‐based treatment did not have improved efficacy compared to acyclovir. Another study has been registered at ICTRP and is currently recruiting, studying the topical effects of Dynamiclear, containing flower extracts of *Hypericum perforatum* and *Calendula officinalis*, and 5% copper sulfate, on herpes simplex labialis.^53^ A previously published clinical study has also investigated a similar Dynamiclear topical formulation containing 0.10% *H. perforatum* and 5% copper sulfate pentahydrate for herpes skin lesions.^54^ In this study, the product was compared against 5% acyclovir cream and disease progression recorded by a clinician—the Dynamiclear group had lower rates of pain and vesiculation than in the acyclovir group; however, the study was not blinded.

Several studies investigating natural compounds against HSV are registered at clinicaltrials.gov. A topical formulation for herpes simplex labialis, an *Azadirachta indica* (neem)‐based lip balm, is being investigated in a clinical trial with no results posted. BOR15001L7, an undisclosed natural compound for the topical treatment of herpes simplex labialis, has a registered trial that is not yet recruiting. Botulinum toxin type A, a protein produced by *Clostridium botulinum*, has been formulated and studied in a trial as an injection for the treatment of herpes simplex labialis, but this trial has been terminated. Another trial studied ZEP‐3 topical 10% ointment for herpes simplex labialis, a peptide derived from an undisclosed species of snakes with no results posted.

In a clinical study, an oral herbal mixture of *Wisteria floribunda*, *Trapa natans*, *Terminalia chebulae*, *Coix lachrymajobi*, *Ganoderma lucidum*, and *Elfuinga applanata* was investigated for the treatment of herpes lesions.^55^ The results showed shortened recovery time in the treated group. This study was not blinded, and furthermore, the control group had no placebo treatment. What parts of the plants that were used for the dried aqueous extract is also not stated. Another clinical study investigated the effects of an oral treatment of a plant and root extract of *Echinacea purpurea* on recurrent genital herpes.^56^ The study was a crossover, double‐blinded placebo controlled trial. There was no difference between the groups in frequency and duration of recurrence, pain, or serological parameters. Saller et al. investigated a topical treatment of *Rheum officinale, Rheum palmatum*, and *Salvia officinalis* root and leaf extracts on herpes labialis.^57^ This treatment was compared to topical acyclovir administration in a randomized single‐blinded trial. There was no statistical significance between the treatments.

Evident from the clinical pipeline of natural products against HSV infections, this approach focuses on wound healing and pain management of the lesions caused by the virus. Furthermore, mostly extracts of natural products are investigated clinically.

### 5.4 Gene editing

The use of targetable gene editing enzymes, for example, homing endonucleases, zinc finger nucleases, transcription activator‐like effector nucleases, and CRISPR/Cas9 (clustered regularly interspaced short palindromic repeats/CRISPR‐associated protein 9), has allowed research aiming at elimination or inactivation of persistent viral DNA genomes in their host cells.

The latent HSV renders itself a suitable target for gene editing due to its presence as an entire viral DNA molecule in the latently infected cell.^58^ However, latent HSV DNA contains complex modifications, the majority of the genome displaying markers of silenced chromatin, while the locus of viral latency‐associated transcription displays markers of active chromatin.^59^ Chromatin modifications of eukaryotic cell genomes greatly affect the efficacy of gene editing.^60^ Designer endonucleases (meganucleases) have been applied for cleavage of resident HSV DNA molecules in cultured cells and in in vivo models.^61^ The target viral genes for the meganucleases have included the HSV UL19 gene, encoding the viral major capsid protein VP5, and the UL30 viral DNA polymerase gene.^61^ ^b^ The transport of the meganucleases to cells or to nerve ganglia has been accomplished using an adeno‐associated virus (AAV) vector, and an additional AAV vector has been utilized for delivery of a 3′‐5′‐exonuclease (three prime repair exonuclease 2) in order to expand the double‐stranded DNA breaks resulting from the meganuclease activity.^61^ ^b^ Chemical inhibition of cellular histone deacetylase has further improved the mutagenesis of resident viral DNA.

In comparison with the CRISPR/Cas9 approach, meganuclease treatment has appeared effective in elimination of latent HSV in vivo in certain loci in the nervous system. In a mouse ocular infection model of HSV latency, the superior cervical ganglia were 90% cleared of reactivatable intact HSV; meanwhile, in the trigeminal ganglia (the principal site of HSV latency in the model) over 50% of the latent viral genomes were affected.^61^ ^c^ AAV‐mediated Cas9‐treatment did not induce remarkable levels of mutations in the HSV DNA, although a novel target in the HSV gene UL54 was applied (coding for the immediate early ICP27 protein).^61^ ^c^ These meganuclease studies did not target the HSV UL29 gene, coding for the single‐stranded DNA binding protein ICP8, which have proven an amenable target for RNA interference‐based treatments.^62^

On the other hand, HSV UL29 and UL8 genes were targeted by CRISPR/Cas9‐treatment utilizing lentiviral delivery of the guide RNAs and Cas9‐coding mRNAs.^63^ Using this approach, HSV‐1 infection was controlled in therapeutic and preventive models of mouse HSV keratitis as well as in cultures of human corneal tissue. However, elimination of reactivatable HSV genomes from trigeminal ganglia by in vivo treatment during viral latency was not an object of the study. Indirectly, reduction of reactivated HSV in the eyes and of HSV DNA load in the trigeminal ganglia resulted from the gene editing treatment delivered after reactivation of the latent virus in the mouse model.^63^ Subsequently, inspired by the findings of the aforementioned study, a clinical trial has been initiated to evaluate the safety, tolerability, and efficacy of gene editing therapy of refractory herpes keratitis (NCT04560790).

### 5.5 Oligonucleotide‐based treatment

Oligonucleotide‐based treatment can exploit various types of DNA or RNA molecules, including short interfering RNA (siRNA), microRNAs (miRNAs), and antisense DNA oligonucleotides (ASOs). All of the aforementioned act via sequence specific recognition of their complementary oligonucleotide sequence. For antiviral therapy, they have wide potential, as viral nucleotide sequences are distinct from those of humans. In general, the antisense oligonucleotide approach (utilizing any antisense oligonucleotide) is extremely straightforward: for initiation of therapy development, only a genomic sequence, nowadays available in days after discovery of a novel pathogen, is required.

The intrinsic regulatory miRNAs of human and of HSV are widely studied for their role in regulation of HSV lifecycle and host antiviral response, but only a few studies have been conducted regarding the potential of miRNAs as antiviral therapy of HSV infection. Nevertheless, recently, both natural and synthetic miRNAs have yielded promising results in vitro in inhibition of HSV replication.^64^

Both ASOs and siRNAs hybridize to their target RNA in a sequence‐specific manner, causing RNA degradation or preventing mRNA processing or translation. To our knowledge, ASOs have not yet been studied as antiviral therapy for HSV, though they have proven efficient in clinical studies against another DNA virus, hepatitis B virus.^65^ Like ASOs,^66^ siRNAs are recognized as having therapeutic potential against emerging pathogens and otherwise untreatable pathogens, including those that have treatments with only limited effectiveness, and those that are in risk of developing drug resistance.^67^ Though no antiviral siRNAs are yet on the market, various antiviral siRNAs have been studied in clinical trials.^67^ Still, no siRNAs targeting herpesviruses have yet reached clinical trials.

siRNAs offer a valuable approach with a novel mechanism of action for the treatment of HSV. The treatment would benefit not only those patients suffering from episodes caused by drug‐resistant strains of HSV, such as numerous patients with herpes keratitis, but also those in need of a more efficient treatment, such as patients with herpes encephalitis. Moreover, as the diseases caused by herpes simplex viruses are most commonly topical (eye, skin, and mucosa), the delivery of siRNA is not as challenging as with intravenous, systemic treatment. As topical treatments lead only to low‐systemic loads of siRNA,^68^ the overall adverse effects could be minimal. Furthermore, intranasal delivery of siRNA could reach the central nervous system^69^ and thus enhance the treatment of herpes simplex encephalitis^70^ or eventually even target the latent or reactivating infection of HSV in sensory ganglia.

Even though siRNAs against HSV are not yet studied in clinical trials, they have been studied in vitro and in vivo with promising results. Of the targets studied, UL39‐ and UL29‐targeted siRNAs have demonstrated efficacy against various patient‐derived strains in vitro,^62^ ^a,^ ^71^ as well as efficacy in vivo,^62^ ^b,^ ^70^ ^b,^ ^72^ encouraging for further research with these targets. Moreover, as UL29 has high homology between HSV‐1 and HSV‐2, siRNAs targeting UL29 have demonstrated in vivo efficacy against both of the viruses.^62^ ^b,^ ^72^ Altogether, in in vivo studies, siRNAs against HSVs have led to decreased symptoms and mortality as well as to significant inhibition of virus production in the infected tissues.^62^ ^b,^ ^70^ ^a,^ ^72^ ^,^ ^73^ The promising preclinical studies suggest that siRNAs are a viable modality for the future prevention and treatment of HSV.

Antiviral siRNAs are challenged with emergence of resistance due to the short target sequence of siRNA. To circumvent the issue of emerging resistance, an enzymatic method of siRNA production was developed, which generates pools of siRNAs (“siRNA swarms”) targeting up to thousands of base pairs of the pathogen sequence, allowing easier target selection, lower possibility for cumulative off‐target effects, and lower possibility of emergence of resistance. The approach has proven efficient in vivo^72^ as well as demonstrated stable antiviral efficacy against a wide range of HSV strains in vitro^62^ ^a,^ ^74^ and against an ACV‐resistant strain^62^ ^a^ without any observed adverse cellular response.^70^ ^b,^ ^75^

### 5.6 Immunomodulatory treatment

Immunomodulatory treatment of HSV infection is of high interest especially as adjuvant therapy in the more severe disease forms, such as herpes encephalitis and herpes keratitis, to prevent exaggerated inflammation and related destruction of the tissues. However, the balance between the antiviral inflammation and exaggerated inflammation is delicate, and the use of immunomodulatory drugs is for that reason somewhat controversial. One of such immunomodulatory treatments is corticosteroids, which have been suggested to be efficient as adjuvant treatments for herpetic diseases^76^; however, their use may be controversial since they modify the immune responses against herpes.^77^ The current pipeline has one corticosteroid, dexamethasone, which has previously improved herpes encephalitis disease outcomes according to many case reports.^76^ ^b^ Dexamethasone is studied in a currently recruiting phase III trial to reduce cognitive defects related to herpes encephalitis (Table 2). Anti‐inflammatory treatment is also studied for less severe disease: a topical diclofenac‐lidocaine mixture (RMN3001) and a squaric acid dibutyl ester (SQX770, SADBE) are studied in clinical trials for herpes labialis‐related pain or recurrences, respectively. SADBE, a suggested immune system enhancer, has been reported to be more effective than the placebo treatment in recurrence prevention.^78^ As suggested by phase I results, its efficacy to prevent recurrences is likely based on induced interferon γ levels and decreased interleukin 5 levels.^79^

## 6 SUMMARY

HSV causes lifelong infections without a sterilizing cure. With the emergence of acyclovir‐resistant strains, the diseases caused by HSV infections are recognized as an unmet medical need. Due to this, several different approaches are utilized in the hopes of finding a therapy that treat infections with a different mechanism of action than the current therapies or to find a cure for latent infections.

The approaches that are being utilized in the clinical pipeline include small molecule inhibitors, gene editing, biopharmaceuticals including therapeutic vaccines, natural products, and immunomodulatory treatment. The compounds with an established mode of action are presented in Figure 2. Furthermore, emerging approaches, such as oligonucleotide‐based treatments, are in preclinical development. These approaches have potential to solve the unmet medical need, including treating infections caused by HSV strains resistant to nucleoside analogs or potentially targeting latent HSV infections, and thus eliminating the latent virus from the host.

## AUTHOR CONTRIBUTIONS

All authors contributed to planning and writing of the manuscript.

## CONFLICT OF INTEREST

The authors declare no conflict of interest.
